# Effects of Creep Feeding from Birth to Suckling Period on Hanwoo Calves’ Growth Performance and Microbiota

**DOI:** 10.3390/ani15152169

**Published:** 2025-07-23

**Authors:** SoHee Lee, Young Lae Kim, Gi Hwal Son, Eui Kyung Lee, Nam Oh Kim, Chang Sik Choi, Kyung Hoon Lee, Hyeon Ji Cha, Jong-Suh Shin, Min Ji Kim, Byung Ki Park

**Affiliations:** 1 Department of Animal Science, Kangwon National University, Chuncheon 24341, Republic of Korea; seesohev@naver.com (S.L.); dudfo123@naver.com (Y.L.K.);; 2 Nonghyup Livestock Research Center, Anseong 17558, Republic of Korea

**Keywords:** Hanwoo, calf, creep feeding, microbiota, growth performance

## Abstract

This study evaluated the effects of early-life creep feeding with a high-protein, high-energy diet on growth performance, ruminal fermentation, and gut microbiota in Hanwoo calves (n = 10). Calves were assigned to control or treatment groups from birth to 6 months of age. No significant differences were observed in body weight, average daily gain (ADG), or feed conversion ratio (FCR), but ADG and dry matter intake (DMI) tended to be higher in the treatment group. Ruminal pH, NH_3_-N, and volatile fatty acid (VFA) concentrations showed no significant differences. Fecal VFA profiles exhibited numerical trends suggesting higher propionate at 3 months and lower acetate, butyrate, and total VFA at 6 months in the treatment group, potentially reflecting altered substrate availability or absorption capacity, though these mechanisms were not directly measured. Microbiota analysis indicated stable ruminal alpha diversity, with numerical increases in fecal *Bacteroidetes* and genera such as *Fournierella* and *Flavonifractor* in the treatment group. These results suggest that early creep feeding with high-nutrition diets can support intake and promote potential shifts in hindgut microbiota composition without compromising overall microbial stability. Further research with larger sample sizes is needed to confirm these trends and assess long-term impacts on calf health and productivity.

## Simple Summary

Creep feeding with high-protein, high-energy diets is used to improve calf growth and gut health during early life. This study examined Hanwoo calves fed such diets from birth to 6 months. While no statistically significant differences were found in growth traits like weight or average daily gain, the treatment group tended to show higher intake and growth rates. Ruminal fermentation measures remained stable without significant changes. Fecal fermentation products and microbiota composition showed numerical trends suggesting subtle shifts with age in the treatment group but overall maintaining stable diversity. These results suggest that early high-nutrition creep feeding can support calf intake and potentially influence gut microbial communities without disrupting microbial balance. Further research with more animals is needed to confirm these trends and evaluate long-term effects on calf health and productivity.

## 1. Introduction

The effects of initial ruminant nutrition strategies on their growth, metabolism, and gut microbiota development are increasingly being studied. The rumen and gastrointestinal tract rapidly develop structurally and functionally during the early growth of calves. The nutritional strategies implemented during this period strongly impact future ruminant productivity and health. Developmental or fetal programming is the theory that the maternal nutritional status and endocrine environment during pregnancy permanently affect the physiological, metabolic, and growth pathways of the offspring [1,2]. Mammalian organ and tissue development continues for a specific period after birth, and various environmental factors during this period can substantially influence the physiological traits and phenotypic outcomes of the offspring [3,4]. Initial nutrition influences organ functions and metabolic pathways in a phenomenon known metabolic imprinting [3].

Hanwoo calves generally obtain sufficient amounts of nutrients from maternal milk to ensure normal growth up to approximately three weeks of age; however, milk production declines after this period, necessitating nutritional supplementation of the calf through concentration feeding. Providing formula feed during this period is crucial in supplying additional energy as well as stimulating microbial activity and ruminal villi development, facilitating the early establishment of ruminant-specific digestive physiology [5]. The initial feeding of high-nutrition diets influences body weight gain, muscle and gastrointestinal tract development, and the expression of metabolic genes [6,7]. The rumen is undeveloped at birth and functionally resembles that of a monogastric animal. The rumen then structurally and functionally matures rapidly during the weaning stage. This developmental process is highly sensitive to environmental factors, particularly nutritional input, which plays a pivotal role in shaping the early establishment and diversity of the rumen microbial community [8,9].

The rumen microbiota plays a central role in carbohydrate and protein metabolism, volatile fatty acid (VFA) production, and immune regulation [10]. The microbial community is established within the first few weeks of life and has long-term effects on the host [11,12]. Therefore, we hypothesized that initially supplementing weaned Hanwoo calves with a high-protein, high-energy diet would promote rumen maturation and affect their growth as well as microbial development. We evaluated the effects of creep feeding on growth performance and rumen microbiota to test this hypothesis. Our findings can be used to guide the development of strategies for improving calf productivity and gut health.

## 2. Materials and Methods

This study’s use of animals and the protocols for this experiment were reviewed and pre-approved by the Institutional Animal Care and Use Committee (IACUC) of Kangwon National University committee (KW-210716-1)

### 2.1. Animals, Treatments, and Management

This study was conducted at the Kangwon National University experimental farm in Chuncheon, Gangwon-do, using ten Hanwoo calves (average birth weight: 30.2 ± 6.6 kg). The calves were assigned randomly to one of two groups: a control group fed a conventional formula feed (CP 17.5%, TDN 69.5%) + annual ryegrass straw, and a treatment group fed a high-nutrition formula feed (CP 21.0%, TDN 75.0%) + annual ryegrass straw During the suckling period, the experimental animals were housed in a space created by combining two adjoining pens (4.0 × 8.0 m each), with five dams housed in shared pen. A separate, calf-only compartment was installed on one side of the pen, allowing only calves to pass through freely. Within this calf area, formula feed, roughage, and water were provided ad libitum.

After the intake of colostrum, calves gradually adapted to formula feed by placing small amounts in their mouths beginning 3 to 4 days after birth as part of the creep feeding strategy. Calves were separated from their dams at 3 months of age. At this time, five calves per treatment group were housed together in a single 4 × 8 m pen per group. Formula feed, roughage, and water were provided ad libitum until 6 months of age. Feed and water were offered at the group level, and therefore the group was considered the experimental unit for statistical analysis. All other management practices followed the standard procedures of the Kangwon National University experimental farm.

The chemical composition of the experimental feed was analyzed according to the methods of the AOAC [13]. Neutral detergent fiber (NDF) and acid detergent fiber (ADF) were determined using the method described by Van Soest [14]. The chemical composition of the experimental feed is presented in Table 1.

### 2.2. Measurement and Sample Collection

#### 2.2.1. Growth Performance

Average daily gain (ADG) of the calves was calculated by measuring body weight at birth, 3 months, and 6 months of age using a digital cow scale. Dry matter intake (DMI) was estimated by subtracting the residual amounts of concentrate and roughage measured before feeding at 08:00 from the total amount offered. Feed intake (dry matter, formula feed, and roughage) was measured at the pen level, and the values were averaged per calf by dividing the total intake by the number of animals in each pen. As a result, these variables were not subjected to statistical analyses involving post hoc comparisons. Feed conversion ratio (FCR) was calculated using the ratio of DMI to ADG. Body measurements were taken using a measuring tape, including wither height, body length, and chest girth.

#### 2.2.2. Microbiome Sampling, Profiling, and Analysis

Rumen fluid and fecal samples were collected prior to the morning feeding during body weight measurements at 3 and 6 months of age. Rumen fluid was obtained using a stomach tube and transferred to 50 mL conical tubes. The samples were immediately snap-frozen in liquid nitrogen and stored at −80 °C until analysis. For fecal samples, approximately 20–30 g of feces was collected directly from the rectum of each animal while wearing sterilized latex gloves and rectal examination gloves. The samples were immediately transferred to sterilized 50 mL conical tubes, and the gloves were replaced after sampling each individual. The fecal samples were snap-frozen in liquid nitrogen and stored at −80 °C until analysis.

DNA was extracted using the QIAamp^®^ Fast DNA Stool Mini Kit (Qiagen, Hilden, Germany) following the manufacturer’s protocol for 16S rRNA gene amplification. The extracted DNA was submitted to Macrogen Inc. (Teheranro 238, Gangnamgu, Seoul, Republic of Korea) for next-generation sequencing (NGS) analysis. The sequencing libraries were prepared according to the Illumina 16S Metagenomic Sequencing Library protocols to amplify the V3 and V4 region. The input gDNA 2 ng was PCR amplified with 5x reaction buffer, 1 mM of dNTP mix, 500 nM each of the universal F/R PCR primer, and Herculase II fusion DNA polymerase (Agilent Technologies, Santa Clara, CA, USA).

The cycle condition for 1st PCR was 3 min at 95 °C for heat activation and 25 cycles of 30 s at 95 °C, 30 s at 55 °C, and 30 s at 72 °C, followed by a 5 min final extension at 72 °C. The universal primer pair with Illumina adapter overhang sequences used for the first amplifications were as follows: V3-F: 5′-TCGTCGGCAGCGTCAGATGTGTATAAGAGACAGCCTACGGGNGGC WGCAG-3′; V4-R: 5′-GTCTCGTGGGCTCGGAGATGTGTATAAGAGACAGGACTACHVGGGTATCTAATCC-3′. The 1st PCR product was purified with AMPure beads (Agencourt Bioscience, Beverly, MA, USA). Following purification, the 2 µL of 1st PCR product was PCR amplified for final library construction containing the index using NexteraXT Indexed Primer. The cycle condition for the 2nd PCR was the same as the 1st PCR condition, except for 10 cycles. The PCR product was purified with AMPure beads. The final purified product was then quantified using qPCR according to the qPCR Quantification Protocol Guide (KAPA Library Quantificatoin kits for IlluminaSequecing platforms, Roche, Wilmington, MA, USA) and qualified using the TapeStation D1000 ScreenTape (Agilent Technologies, Waldbronn, Germany). The paired-end (2 × 300 bp) sequencing was performed by Macrogen using the MiSeq™ platform (Illumina, San Diego, CA, USA).

#### 2.2.3. Bioinformatic Profiling and Downstream Analyses

Raw paired-end reads were demultiplexed and imported into QIIME 2 (version 2023.02) for initial processing. Quality control was performed using the DADA2 plugin to trim primers, filter low-quality bases (Q-score < 25), remove chimeras, and denoise sequences, yielding a high-resolution amplicon sequence variant (ASV) table. Taxonomic profiling of ASVs was carried out with a Naive Bayes classifier trained on the SILVA 138 reference database (99% similarity), and the resulting taxonomy assignments were used to compute alpha diversity indices (Shannon, Simpson, Faith’s PD) and beta diversity metrics (weighted and unweighted UniFrac). Sequence quality was further assessed by examining per-sample read counts, Q30 percentages, and library size distributions to ensure consistency across samples. For genetic (phylogenetic) analysis, phylogenetic trees were constructed using MAFFT alignment and FastTree; tree-based diversity metrics were calculated accordingly. Finally, the functional potential of the microbial communities was inferred with PICRUSt2 (version 2.5.1), mapping ASVs to KEGG Orthology (KO) entries and predicting pathway abundances. Downstream statistical analyses and data visualization were performed in R (version 4.2.2) using the phyloseq, vegan, and ggplot2 packages.

#### 2.2.4. pH

Rumen fluid for pH measurement was collected orally using a stomach tube and transferred to a 50 mL conical tube. The pH was then measured using a pH meter (SevenEasy pH, Mettler-Toledo, Greifensee, Switzerland). Rumen pH was measured twice during the study, at the time of rumen fluid sampling conducted at 3 and 6 months of age.

#### 2.2.5. NH_3_-N

Ammonia concentration was determined according to the method described by Chaney and Marbach [15]. For the ammonia analysis, sample preparation was performed as follows. First, 10 mL of rumen fluid was centrifuged at 1250× *g* for 15 min at 4 °C. Then, 5 mL of the supernatant was collected and mixed with 1 mL of 20% metaphosphoric acid (HPO_3_) and 0.5 mL of saturated HgCl_2_ solution. The mixture was centrifuged again under the same conditions (1250× *g*, 15 min, 4 °C), and 1 mL of the resulting supernatant was transferred to a 1.5 mL microtube and stored at −80 °C until analysis. For ammonia analysis, 0.02 mL of the supernatant obtained from centrifugation, along with NH_3_ standards and blanks, was added to test tubes. Then, 5 mL of phenol color reagent—prepared by dissolving 50 g of phenol and 0.25 g of sodium nitroferricyanide [Na_2_(Fe(CN)_5_NO)·2H_2_O] in 1 L of distilled water—was added to each tube. Subsequently, 1 mL of alkali–hypochlorite solution (prepared by dissolving 25 g of NaOH and 16.8 mL of sodium hypochlorite [4–6% NaCl] in 1 L of distilled water) was added. The reaction mixtures were incubated in a water bath at 37 °C for 15 min to allow color development, followed by dilution with 8 mL of distilled water. Absorbance was then measured at 630 nm using a spectrophotometer (VersaMax, Molecular Devices, San Jose, CA, USA).

### 2.3. Statistical Analysis

All data were analyzed using IBM SPSS Statistics (Version 29.0; IBM Corp., Armonk, NY, USA). Because of the small sample size and non-normal distribution of data, differences between the control and treatment groups were assessed using the Wilcoxon rank sum test. Results are presented as medians with interquartile ranges (IQRs), with significance set at *p* < 0.05.

## 3. Results and Discussion

### 3.1. Growth Performance of Hanwoo Calves

The effects of creep feeding on the body weight, average daily gain (ADG), dry matter intake (DMI), and feed conversion ratio (FCR) of the calves are presented in Table 2. The experimental periods were defined as follows: the first, second, and total periods ranged from birth to 3 months, 3 to 6 months, and birth to 6 months of age, respectively. In the 1st period, average daily gain (ADG) tended to be higher in the treatment group compared to the control group. Similarly, during the 2nd period, both 6-month body weight and ADG exhibited a tendency toward higher values in the treatment group. For the total period, ADG was significantly higher in the treatment group than in the control group (*p* < 0.05). DMI was measured on a group basis, and values were averaged per calf. Given that individual intake data were not available, no post hoc statistical tests were performed.

The effects of creep feeding during the first period on the body measurements of Hanwoo calves at 3 and 6 months of age are presented in Table 3. At 3 months of age, wither height tended to be greater in the treatment group compared to the control group, while body length and chest girth did not differ significantly. No significant differences were observed in any of the body measurements at 6 months of age. In general, calves require the introduction of solid feed around 3 weeks of age, as their nutritional demands are initially met by maternal milk, but nutrient deficiencies may arise due to a decline in milk yield from the dam [5]. The early stimulation of solid feed intake in calves that were provided adequate colostrum and milk increased growth performance and rumen development [16]. Postnatal muscle growth is primarily determined by an increase in muscle fiber size rather than the number of muscle fibers [17]; as such, the nutritional supply during the early developmental stages plays a critical role in muscle growth and development throughout the life of an animal [18]. The numerical increase in wither height in the treatment group at 3 months of age may suggest a potential influence of early high-nutrition feeding on skeletal development. However, no statistically significant differences were observed for most measurements, and the limited sample size may have restricted the detection of subtle treatment effects. Further studies with larger populations are warranted to confirm these findings. As shown in Table 3, there were no statistically significant differences in body measurements between the control and treatment groups at either 3 or 6 months of age.

### 3.2. Gut Microbiota

#### 3.2.1. Rumen and Fecal Fermentation Characteristics: pH, NH_3_-N, and VFA

The effects of creep feeding on ruminal pH are shown in Table 4. The ruminal pH did not differ between the control and treatment groups at 3 and 6 months of age, suggesting that early high-nutrition feeding had a limited impact on ruminal pH. The ruminal pH should be maintained within 6.0 to 7.0 to optimize fiber digestion and microbial activity. Fiber digestibility decreases and the risk of subacute ruminal acidosis increases when the ruminal pH falls below 6.0 [19,20]. Declines in pH are often associated with excessive high-concentrate feeding, abrupt dietary transitions, low fiber intake, and the rapid accumulation of volatile fatty acids (VFAs) and lactic acid after feeding. The ruminal pH values remained within the optimal range (6.0–7.0) in all groups in this study, indicating that a stable ruminal environment was maintained even under high-nutrition feeding.

The effects of creep feeding on ruminal and fecal NH_3_-N concentrations are presented in Table 5. Although not statistically significant, numerically higher ruminal NH_3_-N concentrations were observed in the treatment group at both 3 and 6 months of age. Fecal NH_3_-N concentrations also showed numerical variation between groups without significant differences. In general, ruminal NH_3_-N levels are influenced by various factors including protein solubility, feed characteristics, and the rumen microbial community [21,22]. While the results of this study did not show significant differences, the numerical trend observed in the treatment group is consistent with previous reports suggesting a positive association between dietary crude protein and ruminal NH_3_-N concentrations [23].

The effects of creep feeding on the acetate, propionate, butyrate, and total volatile fatty acid (VFA) concentrations in the rumen and feces of the calves are presented in Table 6. The VFA concentrations of propionate and total VFA in rumen fluid at 3 and 6 months of age were lower in the treatment group than in the control group, but the differences were not statistically significant. VFA concentrations are influenced by production levels and factors such as the absorption and liquid passage rates [24]. High-nutrition feeds have been suggested to enhance ruminal VFA absorption through mechanisms such as in-creased villus development and improved mucosal blood flow. Therefore, the lower VFA concentrations observed in the treatment group may be explained by differences in fermentation substrate composition or possible physiological adaptations such as increased absorption capacity. However, as this study did not directly assess rumen epithelial development, mucosal structure, or VFA absorption rates using histological or marker-based methods, these interpretations remain hypothetical. Further research employing such direct measurements is required to clarify these mechanisms.

The results of fecal VFA analysis did not show statistically significant differences between groups at either 3 or 6 months of age. The propionate concentration tended to be higher in the treatment group compared to the control group at 3 months of age. This numerical trend may reflect greater passage of dietary starch to the hindgut due to incomplete rumen development at this early stage, potentially resulting in increased microbial fermentation in the large intestine [10,12]. Conversely, at 6 months of age, the acetate, butyrate, and total VFA concentrations tended to be lower in the treatment group than in the control group. This result is likely attributable to the enhanced starch and protein digestibility in the rumen that was induced by the high crude protein and high-TDN feed, which reduced the amount of fermentable substrates reaching the hindgut [25,26,27,28]. The microbiota composition analysis revealed a trend in the changes in the fecal VFA concentrations. The relative abundance of *Fournierella*, a key genus associated with propionate and acetate production, was significantly higher in the treatment group than in the controls, according to the fecal next-generation sequencing (NGS) results. *Fournierella* contributes to the production of short-chain fatty acids (SCFAs), and reductions in *Fournierella* abundance were associated with decreased SCFA levels in animals with diarrhea [29]. However, the fecal VFA concentrations tended to be lower at 6 months of age despite the higher relative abundance of *Fournierella* in the treatment group.

This suggests that the changes in microbiota composition alone did not fully explain the observed VFA patterns, and physiological factors such as reduced fermentable substrate availability or increased SCFA absorption in the intestine may have also contributed to the VFA patterns. Therefore, the observed changes in the ruminal and fecal VFA concentrations were likely not the result of differences in VFA production alone but rather reflected the complex interactions among microbial composition, substrate availability, and intestinal absorption dynamics. Further detailed studies are required to elucidate the mechanisms underlying this finding.

In conclusion, ruminal VFA concentrations may remain relatively stable when the diet composition and intake are similar. However, growth-stage-dependent changes in digestive efficiency may affect the supply of fermentable substrates and microbial metabolism in the hindgut. These findings suggest that creep feeding strategies using high-protein, high-energy feed in the initial period may promote propionate production in early stages. However, the reduced flow of fermentable substrates to the hindgut with advancing age and improved digestive efficiency may contribute to lower VFA production in this region. Further studies are needed to directly assess the effects on rumen development, absorption capacity, and epithelial structure.

#### 3.2.2. Rumen Alpha Diversity and Microbiota of Calves

Table 7 provides the results of the effects of creep feeding on the alpha diversity of rumen and fecal microbiota. No significant differences were observed between the treatment groups in the ASVs, Chao1, Shannon, or Gini–Simpson indices in the 3-month-old calves. Although the Shannon index was slightly lower in the treatment group than in the control group, all values remained above three, indicating that the microbial communities maintained an ecologically stable diversity and evenness. A Shannon index < 2 is indicative of low diversity and the dominance of a few taxa [30], which was not observed in this study. The Gini–Simpson index reflects community evenness, with values approaching one representing high diversity and balance. The Gini–Simpson index was above 0.8 in both groups, suggesting that a stable microbial community structure was maintained, regardless of the nutritional imprinting treatment.

The ASVs, Chao1, Shannon, or Gini–Simpson indices did not differ between the groups at 6 months of age. The Shannon index was above 5, and the Gini–Simpson index exceeded 0.89 across both treatments. These findings are consistent with those of Malmuthuge [31], who reported that the microbial communities in young calves tend to stabilize during early life. These findings suggest that adequate nutrition and the gradual development of the gastrointestinal tract help sustain microbial diversity. Moreover, creep feeding from birth to 6 months did not significantly alter the microbial community structure, indicating stable ecological diversity throughout the study period.

**Figure 1:**
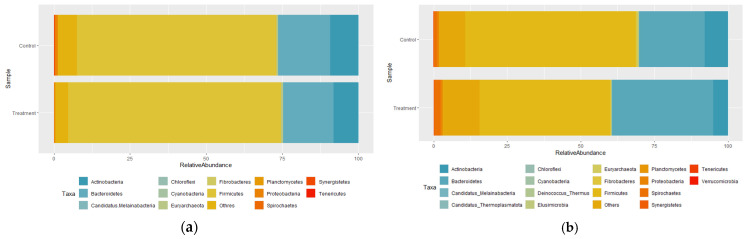
Effect of creep feeding from birth to the suckling period on total microbial population (phylum) in rumen. (**a**) 3 months of age; (**b**) 6 months of age.

The effects of creep feeding during the first period on the rumen microbiota composition of the calves are presented in Table 8 and Figure 1. The dominant phyla in the rumen of 3-month-old calves were *Actinobacteria*, *Bacteroidetes*, and *Firmicutes*, which collectively accounted for over 91% of the relative abundance in the control and treatment groups. The relative abundance of *Firmicutes* was the highest, accounting for more than 65% of the community. *Actinobacteria*, *Bacteroidetes*, *Firmicutes*, and *Spirochaetes* together comprised over 83% of the rumen microbiota at 6 months of age, *Firmicutes* remained the dominant phylum, accounting for more than 44% of the total community. The relative abundance of *Spirochaetes*, which was less than 1% at 3 months, increased to more than 1% at 6 months.

These findings suggest that the first period of creep feeding does not interfere with the normal establishment or development of the major microbial populations in the rumen. Despite administering a high-protein, high-energy feed, the relative abundance of Actinobacteria and Firmicutes decreased and the Bacteroidetes abundance increased in both groups. This pattern is consistent with those found in a previous study, in which Actinobacteria were more prevalent in calves exclusively fed milk, but their abundance declined after introducing solid feed and with increasing age [32]. In contrast, Bacteroidetes is one of the dominant phyla in the preweaning rumen, with an abundance that progressively increases as the calves mature [8]. In addition, the early introduction of solid feed may promote the early establishment of rumen microbial communities, because milk typically bypasses the rumen and flows directly into the abomasum during suckling [33,34]. This suggests that preweaning feeding management has a stronger and longer lasting influence on the microbial composition than postweaning strategies [34]. Differences in preweaning feeding considerably affect the composition of methanogenic archaea after weaning [35] as well as the density of bacteria and protozoa [35]. High-nutrition feed was associated with higher propionate production and lower ruminal pH compared with commercial feed, which correlated with an increased abundance of Proteobacteria [36]. However, no such increase in Proteobacteria abundance was observed in this study. This could be attributed to the ruminal pH in both groups remaining stable within the optimal range of 6.0 to 7.0, and adequate NDF levels being maintained through the concurrent provision of roughage. These results suggest that the inclusion of sufficient forage helps with maintaining rumen environmental stability and suppresses the overgrowth of acidogenic microbial populations even under high-energy and high-protein feeding conditions. In conclusion, creep feeding during the first period did not substantially alter the rumen microbiota composition of the calves; instead, this creep feeding supported the normal development of the dominant microbial taxa and contributed to establishing a stable and healthy gastrointestinal ecosystem. These findings suggest that the nutritional strategies implemented during the early stages of calf management play an important role in promoting long-term gut microbial stability.

The effects of the first period of creep feeding on the rumen microbiota at the genus level are summarized in Table 9, which focuses on the significant taxa and genera of interest based on the study objectives. The relative abundance of *Butyrivibrio* tended to be higher in the control group (5.37%) than in the treatment group (1.82%) in 3-month-old calves. In contrast, the *Prevotella* abundance was similar between the treatment (14.16%) and control (13.41%) groups, suggesting that its abundance was not substantially affected by the dietary intervention. *Butyrivibrio* is a representative butyrate-producing genus in the rumen that contributes to degrading fiber and the production of bacteriocin-like inhibitory substances (BLISs), which suppress competing Gram-positive bacteria and help maintain the microbial balance [37,38]. The observed decrease in *Butyrivibrio* abundance in the treatment group may indicate a corresponding reduction in BLIS production, potentially affecting the competitive microbial dynamics within the rumen. No significant difference was observed in the ruminal acetate concentrations between the treatment and control groups at 3 months of age. This suggested that the decrease in *Butyrivibrio* abundance in the treatment group was functionally compensated by other fiber-degrading microorganisms or that acetate production was not strongly influenced by the changes in specific microbial taxa.

*Prevotella* plays a central role in ruminal carbohydrate as well as hydrogen metabolism and is associated with a stable microbial community structure [39]. Although *Prevotella* degrades polysaccharides via extracellular enzymes and produces propionate and acetate, we found lower concentrations of propionate and acetate in the treatment group despite its higher *Prevotella* abundance.

This discrepancy could be explained by the higher availability of nonprotein nitrogen (NPN) and soluble carbohydrates due to the nutritional imprinting in the treatment group, which led to a metabolic shift favoring microbial protein synthesis over VFA production. Alternatively, enhanced ruminal epithelial development could have promoted increased VFA absorption, resulting in lower residual VFA concentrations. These findings suggest that an increase in *Prevotella* abundance does not necessarily correspond to elevated VFA concentrations under certain nutritional and developmental conditions. The relative abundance of Sinanaerobacter tended to be higher in the control group than in the treatment group at 6 months of age. Although *Prevotella* tended to be more abundant in the treatment groups, the difference was not significant. *Howardella* participates in ruminal nitrogen metabolism via urea degradation. *Howardella ureilytica* converts urea into ammonia, contributing to supplying nitrogen for other rumen microorganisms [40]. *Sinanaerobacter* is a genus that is rarely found in the rumen; its physiological roles remain poorly understood.

These findings suggest that creep feeding during the initial period exerts various effects on ruminal microbial composition, metabolic substrate use, and absorption dynamics. Further investigations using species-level metagenomic profiling and directly assessing VFA absorption are warranted to determine the mechanisms underlying these findings.

#### 3.2.3. Fecal Alpha Diversity and Microbiota

The effects of a first period of creep feeding on the fecal alpha diversity of the calves are presented in Table 10. No significant differences were observed between the control and treatment groups for the ASVs, Chao1, Shannon, or Gini–Simpson indices at either 3 or 6 months of age. The Shannon and Gini–Simpson indices were slightly higher in the treatment group than in the control group at 3 and 6 months. These results suggest that feeding a high-protein, high-energy feed as part of nutritional imprinting positively influences the microbial community diversity and evenness in the gut. The Shannon index exceeded 5, and the Gini–Simpson index was larger than 0.91 in both groups, indicating that the diversity and evenness of the fecal microbial communities remained stable, including under nutritional imprinting conditions. These findings suggested that the first period of nutritional stimulation did not induce abrupt changes in the hindgut fermentation environment and that the microbial community structure developed and remained stable throughout the early growth period.

The fecal microbiota is considered a more reliable indicator of long-term feeding patterns and hindgut fermentation than the rumen microbiota [41]. The nutritional conditions during the initial growth period of a calf play a crucial role in shaping the microbial ecosystem of the gut. The provision of sufficient roughage and high-nutrition formula feed during the suckling period likely helped to maintain the microbial diversity and evenness in this study, preventing the overgrowth of abnormally dominant taxa.

Although the composition of gut microbial communities shifted from before to after weaning, the diversity and evenness of the community generally remained stable [42]. In agreement with these findings, our results suggested that a creep feeding strategy combining an early high-protein, high-energy feed with ad libitum roughage intake helped stabilize the gut microbial ecology and supported the healthy intestinal development and the immune function of the calves.

The phylum-level effects of creep feeding during the first period on the fecal microbiota composition are presented in Table 11. Ten phyla were identified in the feces of calves at 3 and 6 months of age. *Firmicutes*, *Bacteroidetes*, and *Actinobacteria* were the dominant phyla in the control and treatment groups, which accounted for 94.42% and 92.45% of the total relative abundances in the control and treatment groups at 3 months of age, respectively.

*Firmicutes*, *Bacteroidetes*, and *Actinobacteria* accounted for 89.73% and 92.57% of the microbial community in the gut in the treatment and control groups at 6 months, respectively, indicating that the dominant phyla remained stable during the postnatal development period. The effects of nutritional imprinting on the relative abundances of specific phyla were more pronounced. *Bacteroidetes* tended to be more abundant in the treatment group than in the control group at 3 months of age, while *Firmicutes* and *Actinobacteria* showed no statistically significant differences between groups. *Firmicutes* and *Euryarchaeota* were slightly more abundant in the control group, whereas *Bacteroidetes*, *Actinobacteria*, and *Verrucomicrobia* were more abundant in the treatment group at six months, although these differences were not significant.

These findings suggest that the initial period of creep feeding influenced the composition of specific microbial communities in the hindgut. In particular, the increase in Bacteroidetes and the decrease in *Firmicutes* abundance observed in the treatment group reflected the impact of the high-protein, high-energy diet on the intestinal fermentation patterns. *Bacteroidetes* play a key role in fiber degradation and short-chain fatty acid production, with their relative abundance increasing with age [31,43]. In contrast, *Actinobacteria* tend to be more abundant in the early stages of development, but this abundance declines with growth [32]. These transitional patterns were also observed in this study.

The *Euryarchaeota* are a major group of methanogenic archaea in the rumen and hindgut, and their activity is a key contributor to energy loss in ruminants [42]. The relative abundance of *Euryarchaeota* was consistently lower in the treatment group than in the control group at 3 and 6 months of age. This suggested that nutritional strategies during the initial period influenced the initial colonization or activity of methanogenic microbes [44,45,46,47,48]. Our findings suggest that the early nutritional strategy may have the potential to improve energy utilization by modulating methanogenic microbial pathways, as methanogenesis is one of the primary routes through which energy is lost in the rumen. However, as methane emissions or overall energy balance were not directly measured in this study, these interpretations remain speculative and require further validation through direct measurements. In summary, early supplementation with a high-protein, high-energy diet as part of a nutritional imprinting strategy partially influenced the composition of fecal microbial communities at the phylum level in calves, notably inducing changes in the relative abundances of *Bacteroidetes* and *Euryarchaeota*. These findings highlight the potential of nutritional interventions in the initial period to modulate the gut microbiota, which may contribute to improved microbial stability and energy utilization during early growth.

The effects of creep feeding during the initial period on fecal microbiota composition at the genus level are presented in Table 12. Only the genera that significantly differed in abundance between the groups are reported. *Mogibacterium* tended to be more abundant in the control group than in the treatment group at 3 months of age, while *Fournierella* was significantly more abundant in the treatment group. At 6 months of age, *Olsenella* and *Priestia* tended to be more abundant in the control group than in the treatment group. These differences suggested that the initial period of creep feeding induced by the high-protein and high-energy feed influenced the composition of specific gut microbiota genera in Hanwoo calves. *Mogibacterium* is associated with intestinal inflammation and diarrhea [49]. The tendency toward lower relative abundance in the treatment group compared to the control group in this study suggests that early life creep feeding may have contributed to reductions in intestinal inflammatory conditions.

*Flavonifractor* is more abundant in healthy calves, with their abundance being substantially lower in diarrheic individuals [50]. The significantly higher abundance of *Flavonifractor* in the treatment group may suggest a potential link to improved intestinal stability, as this genus is reported to be more abundant in healthy calves. However, we did not directly measure diarrhea incidence or immune parameters in this study, so this interpretation remains speculative. *Fournierella* is another genus that contributes to producing SCFAs. A reduction in *Fournierella* abundance was associated with decreased SCFA concentrations in diarrheic calves [29]. The significantly lower fecal propionate and acetate concentrations in the treatment group (Table 6) could have been linked to the increased relative abundance of *Fournierella*. Although we did not directly assess the incidence of diarrhea in calves, the increased relative abundance of this genus may have influenced intestinal fermentation and microbial metabolite profiles. However, because diarrhea rates and immune markers were not evaluated, further research is required to clarify any potential association between *Fournierella* abundance and gut health outcomes in calves.

*Erysipelothrix*, typically a pathogenic bacterium in humans and other mammals, was detected in the gastrointestinal tract of calves [51] and was identified in this study. The abundance of *Olsenella*, a lactic-acid-producing genus, tended to be lower in the treatment group in 6-month-old calves. An increased abundance of this genus has been associated with diarrhea in calves [52]. *Priestia* belongs to the phylum *Firmicutes* and is involved in degrading protein and carbohydrates in the host [53]. Although data on *Priestia* in ruminants are limited, some human-associated strains degrade mucin, a critical component of the intestinal barrier. An increase in mucin-degrading bacterial abundance can compromise the mucosal defenses and predispose animals to gastrointestinal disorders [54]. *Terrisporobacter* is a Gram-positive spore-forming anaerobe typically found in soil environments [55]. However, the functional roles of *Terrisporobacter* in the growth, maintenance, and pathogenicity in ruminants remain poorly understood. The observed genus-level shifts collectively suggest that creep feeding from birth to 6 months of age directly influences the gut microbial communities of calves. Some of the identified taxa may be functionally linked to fermentation, immune responses, and susceptibility to diarrhea. These findings provide foundational insights for designing early life nutritional strategies to increase calf health and productivity.

## 4. Conclusions

This study evaluated the effects of creep feeding with a high-protein, high-energy diet during the suckling period on growth performance, ruminal fermentation, and gut microbiota in Hanwoo calves. ADG tended to be higher in the treatment group during the pre-weaning periods, with a significant increase observed over the total experimental period. DMI also tended to be higher in the treatment group, despite no statistical tests for intake being performed at the individual level. Ruminal fermentation parameters, including pH, NH_3_-N, and VFA concentrations, showed no statistically significant differences between groups, while fecal VFA concentrations exhibited age-related numerical trends, such as higher propionate at 3 months and lower acetate, butyrate, and total VFA at 6 months in the treatment group. Microbiota analyses demonstrated stable alpha diversity in both rumen and feces, indicating that early high-nutrition feeding did not disrupt overall microbial community structure. However, specific shifts in fecal microbial composition were observed, including a significant increase in *Fournierella* abundance and tendencies toward lower relative abundance of genera such as *Mogibacterium, Olsenella*, and *Priestia* in the treatment group. These changes may be functionally linked to fermentation patterns and potential reductions in intestinal inflammatory conditions. Overall, these findings suggest that early creep feeding with high-protein, high-energy diets can support improved growth performance and induce selective shifts in hindgut fermentation and microbial composition without compromising overall microbial stability. While these results provide preliminary evidence for the potential benefits of early creep feeding strategies, further studies with larger sample sizes, longer follow-up periods, and direct assessments of gut health outcomes are necessary to confirm these effects and evaluate their long-term implications for calf health and productivity. 

## Figures and Tables

**Table 1:** Chemical composition of experimental feed for Hanwoo calves (DM basis).

Items	Control	Treatment	Annual Ryegrass Straw
Dry matter (%)	92.2	92.2	92.0
Crude protein (%)	17.8	21.5	3.2
Crude fiber (%)	7.4	4.2	38.2
Ether extract (%)	2.6	2.8	1.3
Neutral detergent fiber (%)	29.4	21.3	68.1
Acid digestible fiber (%)	14.5	8.9	48.0
Crude ash (%)	7.5	6.4	4.4
Total digestible nutrient (%)	69.9	74.5	50.8

**Table 2:** Effect of creep feeding from birth to the suckling period on growth performance of Hanwoo calves.

Items	Control	Treatment	SEM ^1^	*p*-Value ^2^
1st period				
Birth weight (kg)	28.6	31.7	2.51	0.548
3-month body weight (kg)	86.4	103.5	5.96	0.222
Average daily gain (kg/day)	0.60	0.79	0.06	0.056
Dry matter intake (kg)	0.67	0.78	-	-
Formula feed intake (kg)	0.54	0.64	-	-
Annual ryegrass straw intake (kg)	0.13	0.14	-	-
Feed conversion ratio	1.12	0.99	0.08	0.222
2nd period				
3-month body weight (kg)	86.4	103.5	5.96	0.222
6-month body weight (kg)	178.3	221.0	16.64	0.151
Average daily gain (kg/day)	1.05	1.20	0.07	0.151
Dry matter intake (kg)	4.19	4.51	-	-
Formula feed intake (kg)	3.23	3.49	-	-
Annual ryegrass straw intake (kg)	0.96	1.02	-	-
Feed conversion ratio	3.99	3.76	0.21	0.690
Total period				
Birth weight (kg)	28.6	31.7	2.51	0.548
6-month body weight (kg)	178.3	221.0	16.64	0.151
Average daily gain (kg/day)	0.82 ^b^	1.00 ^a^	0.06	0.032
Dry matter intake (kg)	2.52	2.73	-	-
Formula feed intake (kg)	1.89	2.07	-	-
Annual ryegrass straw intake (kg)	0.63	0.66	-	-
Feed conversion ratio	3.07	2.73	0.39	0.095

Feed intake values were measured per pen and divided by the number of calves. Post hoc tests were not conducted due to the lack of independent replication. ^a,b^ Means in the same row with different superscripts differ significantly (*p* < 0.05). ^1^ SEM: standard error of the mean. ^2^ *p*-Value: *p*-Values were calculated using the Wilcoxon rank sum test (non-parametric).

**Table 3:** Effect of creep feeding from birth to the suckling period on body conformation traits of Hanwoo calves.

Items	Control	Treatments	SEM ^1^	*p*-Value ^2^
3 months of age				
Wither height (cm)	83.25	88.50	1.58	0.057
Body length (cm)	81.75	92.75	4.83	0.486
Chest girth (cm)	103.00	102.75	1.87	1.000
6 months of age				
Wither height (cm)	102.25	106.25	2.00	0.486
Body length (cm)	106.00	113.50	3.33	0.200
Chest girth (cm)	126.75	135.25	2.81	0.114

^1^ SEM: standard error of the mean. ^2^ *p*-Value: *p*-Values were calculated using the Wilcoxon rank sum test (non-parametric).

**Table 4:** Effect of creep feeding from birth to the suckling period on pH in the rumen of calves.

Items	Control	Treatment	SEM ^1^	*p*-Value ^2^
3 months of age	6.06	6.10	0.21	0.686
6 months of age	6.39	6.75	0.31	0.686

^1^ SEM: standard error of the mean. ^2^ *p*-Value: *p*-Values were calculated using the Wilcoxon rank sum test (non-parametric).

**Table 5:** Effect of creep feeding from birth to the suckling period on NH_3_-N in the rumen and feces of calves.

Items	Control	Treatment	SEM ^1^	*p*-Value ^2^
Rumen (ppm)				
3 months of age	31.92	49.62	5.43	0.114
6 months of age	17.37	36.01	7.67	0.200
Fecal (ppm)				
3 months of age	69.2	53.5	13.65	0.686
6 months of age	95.0	107.3	13.20	0.486

^1^ SEM: standard error of the mean. ^2^ *p*-Value: *p*-Values were calculated using the Wilcoxon rank sum test (non-parametric).

**Table 6:** Effect of creep feeding from birth to the suckling period on volatile fatty acid in the rumen and feces of calves.

Items	Control	Treatment	SEM ^1^	*p*-Value ^2^
Rumen				
3 months of age				
Acetate (%, total VFA)	27.03	26.32	2.72	1.000
Propionate (%, total VFA)	23.88	23.24	3.96	0.343
Butyrate (%, total VFA)	5.65	7.03	0.76	1.000
Total VFA (mM)	56.56	56.59	7.21	0.486
6 months of age				
Acetate (%, total VFA)	42.04	31.32	3.51	0.686
Propionate (%, total VFA)	38.21	24.10	3.98	0.686
Butyrate (%, total VFA)	9.78	7.82	1.22	0.886
Total VFA (mM)	90.03	63.24	8.27	0.686
Feces				
3 months of age				
Acetate (%, total VFA)	21.22	31.16	3.41	0.686
Propionate (%, total VFA)	6.52	10.55	1.05	0.486
Butyrate (%, total VFA)	1.65	2.91	0.34	0.486
Total VFA (mM)	29.39	44.63	4.71	0.686
6 months of age				
Acetate (%, total VFA)	37.53	18.36	3.78	0.343
Propionate (%, total VFA)	10.94	7.73	0.97	0.343
Butyrate (%, total VFA)	3.99	1.40	0.49	0.343
Total VFA (mM)	52.46	27.49	5.13	0.343

^1^ SEM: standard error of the mean. ^2^ *p*-Value: *p*-Values were calculated using the Wilcoxon rank sum test (non-parametric).

**Table 7:** Effect of creep feeding from birth to the suckling period on alpha diversity in rumen of Hanwoo calves.

Items	Control	Treatment	SEM ^1^	*p*-Value ^2^
3 months of age				
ASVs	256.750	232.250	48.29	0.686
Chao1	258.188	233.967	48.74	0.686
Shannon	5.520	4.561	0.45	0.343
Gini–-Simpson	0.935	0.839	0.04	0.343
6 months of age				
ASVs	363.250	339.750	63.23	1.000
Chao1	364.000	342.177	63.34	1.000
Shannon	6.151	5.196	0.47	0.343
Gini–Simpson	0.956	0.891	0.02	0.114

^1^ SEM: standard error of mean. ^2^ *p*-Value: *p*-Values were calculated using the Wilcoxon rank sum test (non-parametric).

**Table 8:** Effect of creep feeding from birth to the suckling period on microbiota population (phylum) in rumen of Hanwoo calves.

Items	Control	Treatment	SEM ^1^	*p*-Value ^2^
3 months of age				
*Actinobacteria* (%)	9.23	8.19	2.46	1.000
*Bacteroidetes* (%)	17.12	16.50	5.86	1.000
*Firmicutes* (%)	65.45	69.91	6.49	1.000
6 months of age				
*Actinobacteria*	7.87	5.02	3.37	0.686
*Bacteroidetes*	22.30	34.38	7.63	0.686
*Firmicutes*	57.90	44.29	7.99	0.486
*Spirochaetes*	1.06	2.18	0.71	0.486

Only microorganisms with more than 1% occupancy are shown. ^1^ SEM: standard error of the mean. ^2^ *p*-Value: *p*-Values were calculated using the Wilcoxon rank sum test (non-parametric).

**Table 9:** Effect of creep feeding from birth to the suckling period on microbiota population (genus) in rumen of Hanwoo calves.

Items	Control	Treatment	SEM ^1^	*p*-Value ^2^
3 months of age				
*Butyrivibrio* (%)	5.37	1.82	0.94	0.057
*Prevotella* (%)	13.41	14.16	5.11	1.000
6 months of age				
*Howardella* (%)	0.03	0.00	0.01	0.686
*Sinanaerobacter* (%)	0.18	0.07	0.03	0.057
*Prevotella* (%)	17.07	20.94	6.16	0.114

Only microbiota exhibiting statistically significant (*p* < 0.05) differences or those showing a potential trend toward association are presented in the table. ^1^ SEM: standard error of the mean. ^2^ *p*-Value: *p*-Values were calculated using the Wilcoxon rank sum test (non-parametric).

**Table 10:** Effect of creep feeding from birth to the suckling period on alpha diversity in feces of Hanwoo calves.

Items	Control	Treatment	SEM ^1^	*p*-Value ^2^
3 months of age				
ASVs	328.25	372.50	35.03	0.686
Chao1	333.27	375.73	35.98	0.686
Shannon	5.63	6.38	0.25	0.200
Gini–Simpson	0.913	0.961	0.02	0.343
6 months of age				
ASVs	382.50	380.00	29.17	1.000
Chao1	388.02	385.27	29.67	1.000
Shannon	5.75	6.15	0.34	1.000
Gini–Simpson	0.926	0.951	0.02	0.686

^1^ SEM: standard error of the mean. ^2^ *p*-Value: *p*-Values were calculated using the Wilcoxon rank sum test (non-parametric).

**Table 11:** Effect of creep feeding from birth to the suckling period on microbiota population (phylum) in the feces of Hanwoo calves.

Items	Control	Treatment	SEM ^1^	*p*-Value ^2^
3 months of age				
*Firmicutes* (%)	60.27	47.50	4.75	0.343
*Bacteroidetes* (%)	11.44	31.16	5.41	0.114
*Actinobacteria* (%)	22.71	13.79	5.64	0.343
*Verrucomicrobia* (%)	0.22	1.25	0.41	0.486
6 months of age				
*Firmicutes* (%)	73.13	67.39	6.69	0.886
*Bacteroidetes* (%)	7.80	16.92	4.23	0.200
*Actinobacteria* (%)	8.80	8.26	3.18	0.486
*Verrucomicrobia* (%)	0.21	1.24	0.37	0.486
*Euryarchaeota* (%)	4.27	1.23	1.22	0.200

Marked even if both are above 1%, or one treatment is above 1%. ^1^ SEM: standard error of the mean. ^2^ *p*-Value: *p*-Values were calculated using the Wilcoxon rank sum test (non-parametric).

**Table 12:** Effect of creep feeding from birth to the suckling period on microbiota population (genus) in feces of Hanwoo calves.

Items	Control	Treatment	SEM ^1^	*p*-Value ^2^
3 months of age				
*Mogibacterium* (%)	0.08	0.04	0.01	0.057
*Sinanaerobacter* (%)	0.01	0.10	0.02	0.114
*Flavonifractor* (%)	0.03	0.08	0.01	0.114
*Fournierella* (%)	0.00 ^b^	0.08 ^a^	0.02	0.029
*Erysipelothrix* (%)	0.00	0.02	0.00	0.114
6 months of age				
*Olsenella* (%)	1.50	0.62	0.00	0.057
*Priestia* (%)	0.31 ^a^	0.00 ^b^	0.00	0.029
*Terrisporobacter* (%)	0.09	0.00	0.00	0.114

Only microbiota exhibiting statistically significant (*p* < 0.05) differences or those showing a potential trend toward association are presented in the table. ^a,b^ Means in the same row with different superscripts differ significantly (*p* < 0.05). ^1^ SEM: standard error of the mean. ^2^ *p*-Value: *p*-Values were calculated using the Wilcoxon rank sum test (non-parametric).

## Data Availability

The data are not publicly available due to restrictions imposed by the funding agency. Data may be shared upon request for non-commercial, academic purposes only, and require approval from the corresponding author and the funding institution.
